# Supplementary material for: Establishment of an I-ELISA method based on multi-epitope fusion protein for diagnosis of human brucellosis
Source: PLoS Negl Trop Dis. 2025 Apr 7;19(4):e0012995. doi: 10.1371/journal.pntd.0012995 (PMC12002633; doi:10.1371/journal.pntd.0012995)
Supplement: S1 Text — (DOCX) [file pntd.0012995.s001.docx]

**Establishment of an I-ELISA Method Based on Multi-Epitope Fusion Protein for Diagnosis of Human Brucellosis**

**Yujia Xie^1^** **^¶^ , Liping Guo^1^** **^¶^ , Xinru Qi^1^, Shiqi Zhao^1^, Qichuan Pei^1^, Yixiao Chen^1^, Qi Wu^1^, Meixue Yao^1*^, Dehui Yin^1,2*^**

1. Jiangsu Engineering Research Center of Biological Data Mining and Healthcare Transformation, Xuzhou Medical University, Xuzhou, Jiangsu, China

2. Center for Medical Statistics and Data Analysis, School of Public Health, Xuzhou Medical University, Xuzhou, China

¶ These authors contributed equally to this work

*yaomeixue@163.com (MY); *yindh16@xzhmu.edu.cn (DY)

**Protocols for SAT**

To conduct the experiment, five small test tubes were obtained and arranged on a test tube rack, labeled numerically from 1 to 5. A volume of 2.3 mL of saline was introduced into the first test tube, while the second test tube remained devoid of saline. Subsequently, 0.5 mL of saline was added to each of the third, fourth, and fifth test tubes. A pipette was utilized to transfer 0.2 mL of the sample under examination into the first test tube, ensuring thorough mixing. Following this, 0.5 mL was pipetted from the first test tube and added to both the second and third test tubes, with adequate mixing performed after each addition. Next, 0.5 mL was transferred from the third test tube to the fourth test tube, followed by mixing, and then 0.5 mL was pipetted from the fourth test tube to the fifth test tube, again ensuring proper mixing. Finally, 0.5 mL was pipetted from the fifth test tube and discarded. The resulting dilutions of the samples were calculated as 1:12.5, 1:25, 1:50, and 1:100, corresponding to test tubes 2 through 5, respectively. The reagents were then mixed thoroughly and further diluted tenfold with saline. The first test tube, which contained no antigen, served as a control. Subsequently, 0.5 mL of the diluted reagent was added to each test tube, commencing with the second tube. The final dilutions of the samples were determined to be 1:25, 1:50, 1:100, and 1:200, respectively. The results were interpreted as follows: samples exhibiting a titre of 1:100 or greater were classified as positive for brucellosis.

**Protocols for** **Prokaryotic Expression of Fusion Protein**

A total of 1 μL of recombinant plasmid vector was added to 100 μL of competent cells and the mixture was placed on ice for 30 minutes. Subsequently, a heat shock was applied at 42°C for 90 seconds, followed by immediate placement on ice for 5 minutes. Afterward, 600 μL of LB medium was added, and the cells were incubated at 37°C with shaking at 200 rpm for 40 minutes. Following centrifugation, the entire culture was plated onto LB agar plates containing 50 μg/mL of either Ampicillin or Kanamycin, and the plates were incubated overnight at 37°C in an inverted position. A single colony was then selected from the transformation plate and inoculated into a tube containing 3 mL of LB medium supplemented with 50 μg/mL of Ampicillin. This culture was incubated at 37°C with shaking at 200 rpm overnight. The next day, inoculated 30 mL of LB medium containing 50 μg/mL of Ampicillin or Kanamycin was inoculated at a 1:100 dilution. The culture was then incubated at 37°C with shaking at 200 rpm until the optical density at 600 nm (OD600) reached a range of 0.6 to 0.8. Then, a volume of 1 mL of the culture was removed and subjected to centrifugation at 10,000 rpm for 2 minutes at room temperature, and the supernatant was discarded, the resulting pellet was resuspended in 100 μL of 1× sample buffer, which comprises 50 mmol/L Tris-Cl (pH 6.8), 50 mmol/L DTT, 2% SDS, 0.1% bromophenol blue, and 10% glycerol. Subsequently, isopropyl β-D-1-thiogalactopyranoside (IPTG) was added to the remaining culture to achieve a final concentration of 0.2 mM, thereby inducing fusion protein expression at 37°C with shaking at 200 rpm for 4 hours. Alternatively, IPTG was added to the remaining culture at the same final concentration, and fusion protein expression was induced at 15°C with shaking at 200 rpm overnight. Following this, another 1 mL of the culture was removed, centrifuged at 10,000 rpm for 2 minutes at room temperature, and the supernatant was discarded. The pellet was then resuspended in 100 μL of 1× sample buffer. The remaining culture was centrifuged at 4,000 rpm for 10 minutes, after which the supernatant was discarded, and the pellet was resuspended in phosphate-buffered saline (PBS). After resuspension, ultrasonic cell disruption was performed, and the supernatant and pellet were separated and resuspended in sample buffer. A 12% SDS-PAGE analysis was conducted, with staining using Coomassie Brilliant Blue to visualize the protein bands. Finally, the cell pellet was resuspended in 20 mL of lysis buffer, which consisted of 20 mM Tris-HCl containing 1 mM phenylmethylsulfonyl fluoride (PMSF) and a bacterial protease inhibitor cocktail (pH 8.0), and the cells were disrupted ultrasonically at 400 W power, with a cycle of 4 seconds on and 8 seconds off, for a total duration of 20 minutes. After that, the cell lysate was subjected to centrifugation at 10,000 rpm for 20 minutes at 4°C, and the resulting pellet was collected. The inclusion bodies were washed three times with a wash buffer composed of 20 mM Tris, 1 mM EDTA, 2 M urea, 1 M NaCl, and 1% Triton X-100, adjusted to pH 8.0. Subsequently, the inclusion bodies were dissolved in a solubilization buffer containing 20 mM Tris, 5 mM DTT, 0.15 M NaCl, and 8 M urea, at an appropriate ratio, and incubated at 4°C overnight. Following this, the solution was centrifuged at room temperature at 10,000 rpm for 15 minutes, and the supernatant was collected. The supernatant was then gradually dialyzed against a buffer of 20 mM Tris-HCl and 0.15 M NaCl, pH 8.0, employing a stepwise dilution method while stirring slowly. Finally, the protein solution was placed into a dialysis bag and dialyzed overnight in the same buffer.
