# Supplementary material for: Establishment of an I-ELISA method based on multi-epitope fusion protein for diagnosis of human brucellosis
Source: PLoS Negl Trop Dis. 2025 Apr 7;19(4):e0012995. doi: 10.1371/journal.pntd.0012995 (PMC12002633; doi:10.1371/journal.pntd.0012995)
Supplement: Attachment — Submitted filename: PNTD-D-24-01432_reviewer comments.docx [file pntd.0012995.s003.docx]

Line 55: italicize “Brucella”

Line 66: add “Human” brucellosis to specify host

Line 69: consider changing “yearly” to “annually”

Line 70: consider reordering by relatedness- for example, listing all ruminants together (i.e. sheep, cattle, deer)

Line 71: use lowercase for brucellosis throughout manuscript, except at beginning of sentence

Lines 81-96: add citations throughout this paragraph

Lines 82-83: reorder to bacteriological, molecular, and serological diagnostics to follow flow of paragraph; can also shorten to "molecular" diagnostics/techniques throughout

Line 97: define LPS as an acronym (only define at line 310)

Line 102: change “the” to “a”

Lines 105-108: add citations to support this sentence

Lines 131-137: change tense of paragraph from present to past

Line 143: change “stored in the laboratory” to “used,” acquired,” etc.

Line 150: could shorten genus name to “*B*.”

Line 151: change “types” to “species”

Lines 151-153: this sentence reads vague- if this is pertinent information, please provide additional details or omit

Lines 157-158: are the aa sequences conserved across the three *Brucella* species previously mentioned compared to the outer membrane proteins of other bacterial species? This sentence as written is not clear.

Line 168: For readers unfamiliar with these tools, please clarify what these threshold values mean. Does BCPred not use one? Can rephrase as "we used the default threshold values.." otherwise make past tense.

Line 169-170: this sentence seems redundant- can it be omitted?

Section 2.2.2: change to past tense throughout.

Section 2.2.3: This seems far too detailed a protocol to include in the main methods. Perhaps it could be included as supplementary methods, as desired. Section 2.2.2 may link up with 2.2.4 well as written without even adding a bridging sentence.

Line 253: change “detect” to “test”

Lines 258-261: are the outputs of these t-tests reported in the results? I only see results it reported in Figure 2B but should also be detailed in the main text.

Line 292: adding a percentage could be helpful, i.e. 8 out of 40 serum samples (20%); same with 16 out of 40 with LPS (40%) at line 296- this can help characterize the reduction in cross-reactivity using the fusion protein

Line 295: *P. aeruginosa* is repeated twice- should it be a different bacteria or were there two case of *P. aeruginosa*?

Line 299: change “seen” to "detailed," "provided," etc.

Line 304: are you referring to host or geographic distribution- or both? Specify.

Line 315: change “reducing” to “reduced”

Line 316: “critical for” or “integral to,” as an alternative word choice

Line 336: do you mean “immunity” or “immunogenicity”?

Line 338: did you mean to specify a species of *Salmonella*? If not add *Salmonella “*sp.”

Lines 338-339: it would be helpful to describe why the lack of cross-reactivity with *Y*. *enterocolitica* O9 and *E*. *coli O157:H7* is significant/necessary from a diagnostic application standpoint and why this may supercede cross-reactivity with the other pathogens listed

Line 347: perhaps specify "brucellosis serological testing"

Lines 348-349: if this is specifically calling out the better performance when tested against non-target pathogens, I'm not sure mentioning sensitivity is appropriate here. There may be a more direct way to say this that does not conflate it with the arguably better sensitivity and specificity of LPS compared to fusion protein, when tested on *Brucella* positive or negative sera. I suppose you could also calculate alternate sensitivities and specificities of a combined dataset, where samples are either *Brucella* positive or not, since disease status (negative versus infected with other pathogen) would not be known at the time of initial diagnostic testing. This might be something to consider adding to the results, discussion, or supplemental section.
