# Supplementary material for: Establishment of an I-ELISA method based on multi-epitope fusion protein for diagnosis of human brucellosis
Source: PLoS Negl Trop Dis. 2025 Apr 7;19(4):e0012995. doi: 10.1371/journal.pntd.0012995 (PMC12002633; doi:10.1371/journal.pntd.0012995)
Supplement: Attachment — Submitted filename: Response letter to reviewers.docx [file pntd.0012995.s005.docx]

**Dear Reviewers:**

We really appreciate for your time and comments on our manuscript entitled " Establishment of an I-ELISA Method Based on Multi-Epitope Fusion Protein for Diagnosis of Human Brucellosis" (ID: PNTD-D-24-01432). We have studied all comments carefully and have made corrections. The comments and suggestions provided by the reviewers were constructive, and we implemented the recommended changes. Revised portions are marked in red in the paper. The main corrections in the paper and the responds to the reviewer’s comments are as flowing:

**Methods**

**Response to Reviewer #1:**

Q1: Are the study objectives clearly articulated and a clear testable hypothesis established?
Yes, the authors clearly explain their objective.
**Response:** Thanks for your comment.

Q2- Is the study design adequate to address the stated objectives?
The design is correct and the number of samples is adequate.
**Response:** Thanks for your comment.

Q3- Is the population clearly described and adequate for the hypothesis being tested?
I believe that the authors should better explain the characteristics of the samples (the dates on which they were collected, the tests that were performed, ...).

**Response:** We have added the relevant information we were able to obtain, including collection times and details for SAT (Protocols for SAT have been added in Supporting information 1). At the same time, we are aware that other information about the samples, such as age, sex, and occupation, is necessary for our study; however, since brucellosis is a legally infectious disease in China, this basic demographic information is not allowed to be analysed by us, unless we can obtain authorisation from the Government, the process of this application will be very complicated and lengthy, and it will be very difficult to succeed. So, we beg you to understand that we have not been able to disclose more information about the samples in the manuscript, and we have stated this in the limitations of the manuscript.

Q4- The authors report that the samples are positive for the agglutination test but the gold standard for brucellosis is culture, the authors should explain why they used a serological test as a confirmatory test to evaluate another serological test.
**Response:** At present, the gold standard for the diagnosis of brucellosis is bacterial culture, but bacterial culture requires high laboratory conditions, which grassroots organisations generally do not have. In China, brucellosis is a statutory infectious disease. When hospitals find suspected patients, they send samples to the CDC for confirmation of the diagnosis, and the methods currently used by the CDC are serological methods, including SAT and Rose Bengal Plate Test (RBPT). Therefore, the samples that we have obtained have been confirmed by serological method, but not by culture. Of course, this is indeed a limitation of our research, and we have added relevant statements at the end of the article.

Q5- Is the sample size sufficient to ensure adequate power to address the hypothesis being tested?
Yes,
**Response:** Thanks for your comment.
Q6- Were correct statistical analyses used to support the conclusions?
I felt a lack of statistical comparison between the LPS and fusion protein results.
**Response:** We have added the statistical comparison between the LPS and fusion protein results (Table 2).
Q7- Are there concerns about compliance with ethical or regulatory requirements?
No, all ethical requirements were met.

**Response:** Thanks for your comment.

**Response** **to Reviewer #2:**

Accept with minor revisions as detailed in attachment for clarity.

Q1-Line 55: italicize “Brucella”

**Response:** We have made a correction.

Q2-Line 66: add “Human” brucellosis to specify host

**Response:** We have added it.

Q3-Line 69: consider changing “yearly” to “annually”

**Response:** We have changed “yearly” to “annually”.

Q4-Line 70: consider reordering by relatedness- for example, listing all ruminants together (i.e. sheep, cattle, deer)

**Response:** We've re-categorised and re-ordered them.

Q5-Line 71: use lowercase for brucellosis throughout manuscript, except at beginning of sentence

**Response:** We have carefully checked our manuscript and made corrections, thank you for your suggestions.

Q6-Lines 81-96: add citations throughout this paragraph

**Response:** We have added citations throughout this paragraph.

Q7-Lines 82-83: reorder to bacteriological, molecular, and serological diagnostics to follow flow of paragraph; can also shorten to "molecular" diagnostics/techniques throughout

**Response:** We've reordered to bacteriological, molecular, and serological diagnostics to follow flow of paragraph

Q8-Line 97: define LPS as an acronym (only define at line 310)

**Response:** We've defined LPS, full name of LPS, followed by abbreviation.

Q9-Line 102: change “the” to “a”

**Response:** We've changed “the” to “a”.

Q10-Lines 105-108: add citations to support this sentence

**Response:** We have added citations to support this sentence.

Q11-Lines 131-137: change tense of paragraph from present to past

**Response:** We've changed tense of paragraph from present to past.

Q12-Line 143: change “stored in the laboratory” to “used,” acquired,” etc.

**Response:** We've changed it to “acquired”.

Q13-Line 150: could shorten genus name to “*B*.”

**Response:** We've changed it.

Q14-Line 151: change “types” to “species”

**Response:** We've changed it.

Q15-Lines 151-153: this sentence reads vague- if this is pertinent information, please provide additional details or omit

**Response:** We've deleted this sentence.

Q16-Lines 157-158: are the aa sequences conserved across the three *Brucella* species previously mentioned compared to the outer membrane proteins of other bacterial species? This sentence as written is not clear.

**Response:** Yes, the aa sequences we chose are those that are conserved in *Brucella* species, i.e. not in other bacteria, by BLAST comparison. We have rewritten this sentence and hopefully it will be clear this time.

Q17-Line 168: For readers unfamiliar with these tools, please clarify what these threshold values mean. Does BCPred not use one? Can rephrase as "we used the default threshold values." otherwise make past tense.

**Response:** After carefully checking four tools, we have added an explanation of thresholds and modified the relevant formulation. It is important to note that BCPred is no threshold value.

Q18-Line 169-170: this sentence seems redundant- can it be omitted?

**Response:** We have deleted this sentence.

Q19-Section 2.2.2: change to past tense throughout.

**Response:** We have changed to past tense throughout.

Q20-Section 2.2.3: This seems far too detailed a protocol to include in the main methods. Perhaps it could be included as supplementary methods, as desired. Section 2.2.2 may link up with 2.2.4 well as written without even adding a bridging sentence.

**Response:** We have moved this section to Supporting Information.

Q21-Line 253: change “detect” to “test”

**Response:** We have changed “detect” to “test”

Q22-Lines 258-261: are the outputs of these t-tests reported in the results? I only see results it reported in Figure 2B but should also be detailed in the main text.

**Response:** We have added these t-tests in section 3.3 Evaluation of the Effect of I-ELISA for Detecting *Brucella* and added Table 2 to illustrate these results.

Q23-Line 292: adding a percentage could be helpful, i.e. 8 out of 40 serum samples (20%); same with 16 out of 40 with LPS (40%) at line 296- this can help characterize the reduction in cross-reactivity using the fusion protein

**Response:** Thank you for your suggestion, and we have added the percentage.

Q24-Line 295: *P. aeruginosa* is repeated twice- should it be a different bacteria or were there two case of *P. aeruginosa*?

**Response:** Sorry, due to our oversight, 1 case of Pseudomonas putida was incorrectly written as Pseudomonas aeruginosa, which we have corrected.

Q25-Line 299: change “seen” to "detailed," "provided," etc.

**Response:** We have changed “seen” to "detailed".

Q26-Line 304: are you referring to host or geographic distribution- or both? Specify.

**Response:** Geographic distribution, we have specified.

Q27-Line 315: change “reducing” to “reduced”

**Response:** We have changed “reducing” to “reduced”.

Q28-Line 316: “critical for” or “integral to,” as an alternative word choice

**Response:** We have changed “significant for” to “critical for”.

Q29-Line 336: do you mean “immunity” or “immunogenicity”?

**Response:** After carefully analyzing the relevant reference, we have changed “immunity” to “immunogenicity”.

Q30-Line 338: did you mean to specify a species of *Salmonella*? If not add *Salmonella “*sp.”

**Response:** We have added “spp.”.

Q31-Lines 338-339: it would be helpful to describe why the lack of cross-reactivity with *Y*. *enterocolitica* O9 and *E*. *coli O157:H7* is significant/necessary from a diagnostic application standpoint and why this may supercede cross-reactivity with the other pathogens listed

**Response:** We have carefully reviewed the relevant literature and explained (lines 321-326).

Q32-Line 347: perhaps specify "brucellosis serological testing"

**Response:** We have specified "brucellosis serological testing"

Q33-Lines 348-349: if this is specifically calling out the better performance when tested against non-target pathogens, I'm not sure mentioning sensitivity is appropriate here. There may be a more direct way to say this that does not conflate it with the arguably better sensitivity and specificity of LPS compared to fusion protein, when tested on *Brucella* positive or negative sera. I suppose you could also calculate alternate sensitivities and specificities of a combined dataset, where samples are either *Brucella* positive or not, since disease status (negative versus infected with other pathogen) would not be known at the time of initial diagnostic testing. This might be something to consider adding to the results, discussion, or supplemental section.

**Response:** Firstly, I would like to express my gratitude for your insightful comment. In response to your suggestion, I consulted several colleagues who specialize in statistics. They collectively opined that the inclusion of 40 sera infected with other pathogens in the negative samples for the ROC curve analysis would be inappropriate for two primary reasons. First, these sera are highly selective, and their inclusion would introduce significant selection bias into the ROC curve analysis. Second, the 40 sera in question were not sourced from a homogeneous population; they were collected in 2020 with the assistance of the First Affiliated Hospital of Jilin University, and thus could not be combined in a blind manner. This lack of homogeneity could adversely affect the validity of the statistical analysis. We acknowledge your point regarding the non-representative nature of our samples, and as such, we have incorporated a statement addressing these limitations in the discussion section (lines 358-364).

**Response to Reviewer #3:**

Q1: methods do not state how OD values were used for the "ROC curve analysis" and area under the curve analysis. This undermines the statistical analysis and interpretation for their main findings for iELISA specificity and accuracy conclusions.
**Response:** Because ROC curve analysis is more commonly used in epidemiology and statistics, we have not presented them in detail. To assess the specificity and accuracy of the iELISA method, we employed ROC curve analysis. Specifically, we determined the OD450 values for each sample (Measurements were repeated 3 times for each sample, as accounted for in the Methods section.), the average OD450 value for each sample is then calculated. These OD450 values were then used to calculate the number of true positives (TP), false positives (FP), true negatives (TN), and false negatives (FN). These classifications were based on a predetermined cutoff value established through ROC curve analysis. The cutoff value is the point that maximizes both the sensitivity and specificity, also known as the Youden index (sensitivity + specificity - 1). Based on these classifications, we constructed the ROC curve and assessed the diagnostic performance of the assay by calculating the area under the ROC curve (AUC). An AUC value close to 1 indicates a test with high diagnostic accuracy, while an AUC value close to 0.5 indicates no diagnostic ability. Additionally, we calculated the positive predictive value (PPV) and negative predictive value (NPV), which represent the proportion of true positives among all positive results and the proportion of true negatives among all negative results, respectively.
Data sets of ROC curve analysis using GraphPad Prism 9.5.0 software as follows: Control values, Average OD_450_ values of brucellosis Negative samples; Patient values, Average OD_450_ values of brucellosis Positive samples.

We have added key information on how to use OD450 values for ROC analysis (line lines 221-224, 231-232, 237-240).

Q2: Source of LPS used as control is not stated.

**Response:** We have added relevant information.

**Results**

**Response to Reviewer #1:**

Q1-Does the analysis presented match the analysis plan?
The results lack a statistical complement to compare the obtained results.
**Response:** We have added the results of t-tests in section 3.3 Evaluation of the Effect of I-ELISA for Detecting *Brucella* and added Table 2 to illustrate these results.

Q2-Are the results presented clearly and completely?
Yes, the results are clear but I feel a lack of more statistical comparisons between the two groups evaluated.
**Response:** As stated above, we have added these t-tests in section 3.3 Evaluation of the Effect of I-ELISA for Detecting *Brucella and added Table 2 to illustrate these results.*

Q3-Are the figures (tables, images) of sufficient quality to be clear?
Yes,

**Response:** Thanks for your comment.

**Response to Reviewer #2:**

Q1- Minor revisions: The authors should include results of the independent t-tests outlined in the methods and presently only presented in Figure 2B.

**Response:** We have added these t-tests in section 3.3 Evaluation of the Effect of I-ELISA for Detecting *Brucella* and added Table 2 to illustrate these results.

**Response to Reviewer #3**:

Q1-Unable to safely interpret data without explaining how the OD readings go into the diagnostic curve (AUC) and ultimately into the values for prediction (TABLE 2).
**Response:** As stated above, we have added key information on how to use OD450 values for ROC analysis (line lines 221-224, 231-232, 237-240)..
Q2-I do not understand how data goes into creating Figure 1 panel A.

**Response:** There is no Panel A in Figure 1, and in conjunction with your previous question, we think you mean Panel A in Figure 2. Again, we have already explained how OD450 performs ROC curve analysis.
Q3-It would also appear that the LPS only has significant activity as bait for their iELISA, if not more so than their fusion protein.

**Response**: We agree that LPS showing significant activity in iELISA. However, our fusion protein offers specific advantages. It is designed for low cross-reactivity, enhancing diagnostic specificity, which is crucial for accurate disease identification. While LPS may be more active, our fusion protein provides a safer, more cost-effective, and specific alternative. We are committed to further optimizing our fusion protein to match or surpass LPS's performance in future studies. We've added a related discussion (lines 334-350).

**Conclusions**

**Response** **to Reviewer #1**:

Q1-Are the conclusions supported by the data presented?
Yes, the conclusions are clear and supported by the results.
**Response:** Thanks for your comment.

Q2-Are the limitations of analysis clearly described?
I did not find a paragraph explaining the limitations of the study. The authors could add a paragraph explaining the limitations of the study.
**Response:** We have added the limitations of our study(lines 358-364).
Q3-Do the authors discuss how these data can be helpful to advance our understanding of the topic under study?
The authors need to go more into the benefits of using fusion protein, I feel they can better explain the benefits of low cross-reactivity.
**Response:** We have revised the discussion section to reflect this finding and have explored the underlying reasons for this change (lines 334-350).

Q4-Is public health relevance addressed?
Yes, the authors explain well the importance of looking for other diagnostic options for brucellosis.

**Response:** Thanks for your comment.

**Response to Reviewer #2:**

Q1-Accept, with minor revisions as suggested in attachment.

**Response:** Thanks for your comment. We have revised as suggested in attachment.

**Response to Reviewer #3:**

Q1- Unable to interpret accuracy of the conclusion without logic behind calculations.

**Response:** Based on your comments, we have engaged a statistical professional to evaluate the statistical methods employed in our paper. As you indicated, our initial description of these methods may have been overly simplistic. Consequently, we have made the necessary revisions in light of your previous inquiries.

**Editorial and Data Presentation Modifications?**
Use this section for editorial suggestions as well as relatively minor modifications of existing data that would enhance clarity. If the only modifications needed are minor and/or editorial, you may wish to recommend “Minor Revision” or “Accept”.

**Response to Reviewer #1:**

Q1 -Line 39: write what LPS stands for

**Response:** We have revised it.
Q2-Line 116 - 123: write the full name of the acronym

**Response:** We have added the full name of the acronym**.**
Q3-Line 139: Explain the characteristics of the samples better (on what dates were they collected, tests that were performed to confirm the disease, ...)

**Response:** As noted above, we have added the relevant information we were able to obtain, including collection times and details for SAT. At the same time, we are aware that other information about the samples, such as age, sex, and occupation, is necessary for our study; however, since brucellosis is a legally infectious disease in China, this basic demographic information is not allowed to be analyzed by us, unless we can obtain authorization from the Government, the process of this application will be very complicated and lengthy, and it will be very difficult to succeed. So, we beg you to understand that we have not been able to disclose more information about the samples in the manuscript, and we have stated this in the limitations of the manuscript.

Q4-Line 140: Do the authors consider it appropriate that the confirmatory method for positive samples be agglutination? Since a serological method is being evaluated and the confirmatory test for positive samples is another serological method, other confirmatory tests are needed for positive samples.
**Response:** At present, the gold standard for the diagnosis of brucellosis is bacterial culture, but bacterial culture requires high laboratory conditions, which grassroots organisations generally do not have. In China, brucellosis is a statutory infectious disease. When hospitals find suspected patients, they send samples to the CDC for confirmation of the diagnosis, and the methods currently used by the CDC are serological methods, including SAT and Rose Bengal Plate Test. Therefore, the samples that we have obtained have been confirmed by serology, but not by culture. Of course, this is indeed a limitation of our research, and we have added relevant statements at the end of the article.

Q5-Line 274: Perform statistical tests to compare the two groups and then discuss those results.

**Response:** We have added these t-tests in section 3.3 Evaluation of the Effect of I-ELISA for Detecting *Brucella* and added Table 2 to illustrate these results, and added the discussion.
Q6-Line 296 - 300: the authors explain the cross-reactivity with 7 pathogens but in the discussion they do not explain why this could occur. Does the fusion protein sequence have similarity with the sequence of these pathogens?

**Response:** We have added the discussion (lines 342-347).
Q7-Did the authors perform any analysis of the sequences?
In the discussion I consider it important to include a paragraph about the limitations of the study.

**Response:** We have added the limitations of our study(lines 358-364).

**Response** **to Reviewer #2**:

Q1- Minor revisions- see comments by line number in attachment.

**Response:** We have made point-to-point revisions to our manuscript based on the attached comments.

**Response** **to Reviewer #3:**

Q1- tense of methods section detailing cloning of fusion construct is written as a protocol (do this then do that).

**Response:** We have optimized the language presentation of this section as you suggested, and we have moved this section to Supporting Information as suggested by another reviewer.

Q2-figure legends lack any informative detail.

**Response:** We've rewritten the figure legends to include more information

**Summary and General Comments**

Use this section to provide overall comments, discuss strengths/weaknesses of the study, novelty, significance, general execution and scholarship. You may also include additional comments for the author, including concerns about dual publication, research ethics, or publication ethics. If requesting major revision, please articulate the new experiments that are needed.

Reviewer #1: (No Response)

**Response to Reviewer #2:**

Q1-I think this was a thoughtful, well executed study that draws appropriate conclusions from the results showing that the designed fusion protein can greatly reduce cross-reactivity (false positive rate) compared to the current method using LPS for serological testing. All suggested revisions are fairly minor but meant to tighten up the manuscript and perhaps help make the findings more compelling. I do think it would be interesting to rerun the ROC curve analyses grouping the Brucella-negative sera and sera used to compare cross-reactivity with other infections to consider how the fusion protein compares to LPS under "real world" conditions when disease status is not known prior. Otherwise, I think this is an important study with immediate relevancy and public health application.

**Response:** Firstly, thank you very much for your comments. As noted above, I consulted several colleagues who specialize in statistics. My colleagues think adding 40 samples with other infections to the negative group in the ROC analysis is wrong. It's because: 1) These samples are very specific and will cause bias. 2) They're not from the same group and were collected in 2020, so mixing them could mess up the stats. We know our samples aren't perfect and stated these limitations in the discussion section.

**Response to Reviewer #3:**

Q1- methods do not state how OD values were used for the "ROC curve analysis" and area under the curve analysis. This undermines the statistical analysis and interpretation for their main findings for iELISA specificity and accuracy conclusions.
**Response:** As noted above, we have made the relevant changes as you suggested.
Q2-Source of LPS used as control is not stated.
**Response:** We have added the source of LPS.
Q3-Unable to interpret accuracy of the conclusion without logic behind calculations.

**Response:** As noted above, based on your comments, we have engaged a statistical professional to evaluate the statistical methods employed in our paper. As you indicated, our initial description of these methods may have been overly simplistic. Consequently, we have made the necessary revisions in light of your previous inquiries.

Once again, thank you very much.

Best wishes,

Dehui Yin
